# The potential of *Sonneratia caseolaris* mangrove leaves extract as a bioactive food ingredient using various water extract

**DOI:** 10.12688/f1000research.143708.4

**Published:** 2025-01-03

**Authors:** Hartati Kartikaningsih, Nur Fitriana, Ike Listya Anggraeni, Bambang Semedi, Maharani Pertiwi Koentjoro

**Affiliations:** 1 Fisheries Product Technology Study Program, Faculty of Fishery and Marine Science, Universitas Brawijaya, East Java, 65145, Indonesia; 2 Postgraduate School of Universitas Brawijaya, Universitas Brawijaya, Malang, East Java, 65145, Indonesia; 3 Research Center for Pharmaceutical Ingredients and Traditional Medicine, National Research and Innovation Agency (BRIN), Cibinong Bogor, West Java, 16911, Indonesia

**Keywords:** Sonneratia caseolaris, mangrove leaves, bioactive, antioxidant, food ingredient

## Abstract

**Background:**

*Sonneratia caseolaris*, has been widely utilized by the Indonesian. *S. caseolaris* leaves contain various active compounds, contributing to their popularity in the treatment of various diseases. Mangrove leaves are also known to exhibit very high antioxidant activity. This study aims to assess the antioxidant activity of *S* *. caseolaris* leaves extracted using different solvents. The resulting extract was evaluated for antioxidant activity by the 2,2-diphenyl-1-picrylhydrazyl radical scavenging activity (DPPH) techniques.

**Methods:**

Analysis of total flavonoids, total phenols, identification of active compounds with Liquid Chromatography High Resolution Mass Spectrometry (LC-HRMS), and bioinformatics were also carried out to obtain temporary conclusions about the antioxidant activity of *S. caseolaris* leaf extract.

**Results:**

The results indicated that *S. caseolaris* leaves extracted with methanol and distilled water exhibited the highest antioxidant activity compared to other extracts. The analysis of total flavonoids and total phenols yielded results consistent with the antioxidant activity tests. LC-HRMS results identified three compounds in all *S. caseolaris* leaf extracts with antioxidant activity, namely TEMPO, Choline, and Betaine. TEMPO demonstrated a higher antioxidant activity than Choline and Betaine, as indicated by the binding affinity values in the bioinformatics analysis.

**Conclusions:**

It is evident that *S. caseolaris* leaf extracts has the potential to serve as an effective antioxidant agent. Further research is needed to confirm how the potential compounds in *S. caseolaris* leaf water extracts interact with the target protein Keap1. This research aims to utilize *S. caseolaris* as active components in food products, thereby enhancing antioxidant consumption among consumers.

## Introduction

Mangrove are vegetation community that has high morphological and physiological adaptations and are widely distributed along coastal and brackish swamps in tropical, subtropical, to temperate regions. ^1^ They are highly productivity and provide various ecosystem services for the environment and society. Physically and ecologically, mangroves function as protectors of coastal areas against rising sea levels, abrasion, waves, and storms. ^2^ Biologically, mangrove ecosystems provide breeding and feeding grounds for marine biota. ^3^^,^^4^ Additionally, mangrove ecosystems also function as pollutant filters, able to reduce carbon emissions so that they have the potential to mitigate climate change. ^5^^,^^6^ Economically, mangrove ecosystem serves as a renewable resource for timber and tourism. ^7^ Indonesia has the largest mangrove ecosystem in the world, which is around 26-29% of the world’s mangrove ecosystems. ^8^ Several types of mangroves can be found in Indonesia, including *Sonneratia caseolaris*, *Avicennia marina*, *Rhizophora mucronata*, and *Rhizophora apiculata.* ^9^^,^^10^

*S. caseolaris* is a mangrove species from the Sonneratiaceae family. It typically reaches a length ranging from 5-15 meters and is equipped with respiratory roots. Its flowers are characterized by the presence of many stamens. ^11^^,^^12^ *S. caseolaris* contains various active compounds, including flavonoids, phenolics, alkaloids, terpenoids, tannins, and saponins. ^13^^,^^14^ The presence of these active compounds makes this mangrove species highly sought after for its potential as an antioxidant agent, ^9^^,^^15^ antibacterial, ^9^^,^^14^^,^^15^ anticytotoxicity, ^15^ anti-allergy and antidiabetic. ^16^ *S. caseolaris* leaves, in particular, are recognized for their high potential as an antioxidant agent. ^9^

Antioxidants are natural or artificial compounds that function to scavenge free radicals in the body. Antioxidants can be divided into endogenous antioxidants and exogenous antioxidants. Endogenous antioxidants are types of antioxidants that already exist in the body, while exogenous antioxidants are antioxidants obtained from outside the body, such as antioxidants obtained from food consumption. An imbalance between free radicals and antioxidants in the body can lead to damage and various unwanted effects, including inflammation, aging, and certain diseases, such as cancer and diabetes mellitus. ^17^

Kelch-like ECH-associated protein 1 (Keap1) is a protein that plays a crucial role in reducing endogenous antioxidant activity. The Keap1 protein functions as an endogenous inhibitor of erythroid nuclear factor 2-related factor 2 (Nrf2). When Nrf2 is inhibited, it cannot enter the cell nucleus to induce the expression of antioxidant enzymes. Under abnormal conditions, oxidative stress increases and leading to various damages in the body. ^18^^–^^23^ Keap1 and Nfr2 lead to ubiquitination, resulting in the mimicry of Nfr2 degradation. The degradation of Nfr2 diminishes the regulation of antioxidant response elements when oxidative stress occurs. Therefore, this study hypothesizes that the antioxidants present in *S. caseolaris* may have the potential to bind to Keap1, potentially allowing Nrf2 to function effectively within the cell. This hypothesis is based on the known interactions between similar antioxidants and Keap1 in other studies, although further research is needed to confirm this specific interaction. The effectiveness of *S. caseolaris* antioxidants in binding to Keap1 is crucial for maintaining Nfr2 functionality. By preventing the degradation of Nfr2, these antioxidants ensure that the regulatory mechanisms for antioxidant response remain intact even under conditions of oxidative stress. This mechanism is vital for cellular defence against oxidative damage and underscores the potential therapeutic significance of *S. caseolaris* in combating oxidative stress-related disorders.

In food technology, antioxidants are added as bioactive food ingredients with the aim of improving food quality and consumer health. Previous research has shown that *S. caseolaris* leaves extracted with organic solvents such as ethanol and methanol exhibit high antioxidant activity. However, these solvents are toxic and can leave chemical residues in the extract, which can be dangerous when consumed. In this study, we had to evaluate the antioxidant activity of S *. caseolaris* leaves extracted with distilled water and various types of bottled drinking water. We aim to enhance our analysis to predict how potential compounds in *Sonneratia caseolaris* leaves water extract interact with the target protein Keap1. I has potential compounds interact with a target protein like Keap1, is crucial for understanding their potential therapeutic potential.

## Methods

### Sampling method

The leaves of *S. caseolaris* were collected from a mangrove plant in Ujung Pangkah Gresik, East Java, Indonesia (6.8899940° S, 112.6081658° E). Sample was collected from the third to the fifth leaf, counted from the top of each plant. These leaves were thoroughly rinsed to remove salt using running water and then air-dried at room temperature for 7 days. Subsequently, the dried leaves were mashed into a fine powder for extraction.

### Sample extraction

The extraction process utilized various solvent, including distilled water (A), bottled drinking from three different brands (Ax (B), Cx (C), Kx (D), and methanol (Cat. 179337, Sigma-Aldrich ^®^) (E) for control purposes. The bottle drinking water (B, C, and D) was sourced from the Indonesian market. and commonly used as a type of mineral water in Indonesia. Mangrove leaf powder was extracted with each solvent at ratio 1:3 (w/v). The extraction was conducted through maceration at room temperature for 24 hours, followed by filtration using Whatman ^®^ quantitative filter paper, ashless, Grade 42 (Cat. WHA1442041, Merck). The extraction process was repeated three times for each solvent. The resulting filtrates were subsequently evaporated using a rotary evaporator (DLab rotary evaporator RG100-S, 40°C). Every 1000 grams of fresh leaves will become 200 grams of dried leaves. In this study, the third and fifth leaves are selected based on previous research findings (unpublished data). These leaves have shown higher total phenol, total alkaloid, and flavonoid content compared to other parts of the leaf. However, there is no significant difference in pH and viscosity.

### Calculation of extraction yield and pH examination

The extraction yield was calculated by the following formula. ^1^ The pH of the extracts was measured with a pH meter (Toledo S-220-KIT). The analyzed sample is a working solution derived from the evaporator, consisting of 1 mL of solution mixed with 20 mL of double-distilled water.

$$\text{Yield}=\frac{\text{extract weight}}{\text{leaf powder weight}}\;\times \;100\%$$ [1]

### Total phenol content

The total phenol test refers to Ref. 23 Total phenol content was measured by mixing 30 μL Folin-Ciocalteu (Cat. F9252, Sigma-Aldrich ^®^) 1.0 N reagent with 60 μL of the sample, placed on the microplate. Afterwards, 150 μL of 20% sodium carbonate (Cat. 223530, Sigma-Aldrich ^®^) solution was placed on a microplate, incubated for 15 minutes at room temperature and in a dark place. Samples were centrifuged for 8 minutes at 1600 rpm. The supernatants were measured on a Microplate reader at a wavelength. The total phenol was calculated using the gallic acid (Cat. 91215, Sigma-Aldrich ^®^) standard curve (mgGAE/100g).

### Total flavonoid content

1 mL sample was mixed with 3 mL of ethanol 96% (Cat No. 159010, Sigma-Aldrich ^®^), 0.2 mL of 10% aluminium chloride (Cat. 8.01081, Sigma-Aldrich ^®^), 0.2 mL of 1 M potassium acetate (Cat No. 1.04820, Sigma-Aldrich ^®^), and 5.6 mL of distilled water. The mixture was incubated for 10 minutes at room temperature. The supernatant was measured on a Microplate reader at a wavelength of 415 nm. The total phenol was calculated using the standard curve of quercetin acid (mgQE/100g) (Lerck, Cat No. PHR1488).

### Antioxidant activity

Antioxidant activity was measured using a microplate reader (96-flat bottom with lid). ^23^ The DPPH (Cat. 300267, Sigma-Aldrich ^®^) reagent was prepared at a concentration of 0.2 mM in absolute ethanol. The first column was filled with 200 μL of ethanol as a blank. Line A was filled with sample stocks. 100 μL of ethanol solvent was added to each well plate for serial dilution, except for line A. The second -11 ^th^ column in line A was filled with 200 μL of sample stock solution. Serial dilution was carried out by taking 100 μL from line A, and placing it in lines B, C, D, E, F, and G respectively. In line G, 100 μL solution was removed, resulting in each well containing 100 μL of the solution. Subsequently, 100 μL of DPPH was introduced into each well. Line H was negative control (100 μL solvent mixed with 100 μL of 0.2 mM DPPH reagent). The plate was covered and incubated at room temperature for 30 minutes. All samples were measured at 517 nm wavelength. DPPH absorption inhibition was calculated with the following formula. ^2^

$$\text{Antioxidant Activity}\;\left(\%\right)=\frac{\left(\text{blank}\;\mathrm{abs}.-\text{sample}\;\mathrm{abs}.\right)}{\text{blank absorbance}}\times 100$$ [2]

In order to obtain the percentage of inhibition (Inhibitory Concentration value, IC _50_), a linear regression curve (Y=ax + b) was made with the x-axis as the concentration (μg/mL) and the y-axis as the percentage of inhibition. The IC _50_ value is a calculation of how much of the sample concentration is needed to inhibit 50% of free radical activity.

### Statistical analysis

The data were statistically tested using a completely randomized design with three replications. The normality of the data was tested using the Kolmogorov-Smirnov test and then subjected to one-way ANOVA. Differences between means were determined using Duncan’s multiple range test, with P<0.05 regarded as significant. Statistical analysis was conducted using SPSS version 17.0.

### Detection of active compound using LC-HRMS

LC-HRMS used HPLC (Thermo Scientific Diorex Ultimate 3000 RSLCnano, Japan) with solvent A (0.1% formic acid in water) and solvent B (0.1% formic acid in Acetonitrileà Acetonitrile), column Hypersil GOLD, 5 μm, 150 × 4.6 mm, flow rate 40 μL/min, column temperature 30°C. Mass Spectrometer used Scientific Q Exactive™ with software compound discovery with mzCloud™ MS/MS library. Samples were diluted to a volume of 1500 μL, and vortexed at 2000 rpm, for 2 min. The supernatant was filtered with a 0.22 μm syringe filter. The vial was inserted into the LC-HRMS autosampler.

### Ligand and protein preparation

This study used three compounds that had been identified through LC-HRMS analysis, namely Betaine (CID 247), Choline (CID 305), TEMPO (CID 549976), and one control compound (CID 135263934) which acts as the original inhibitor of the Keap1 protein. Ligand was obtained from the PubChem web server ( https://pubchem.ncbi.nlm.nih.gov/) in Sybil Data Files (SDF) format. Betaine, Choline, and TEMPO are compounds that are known to have antioxidant activity. In this study, it is anticipated that they may bind to Keap1 (6TYP), which serves as an endogenous Nrf2 inhibitor. as an endogenous Nrf2 inhibitor. The Keap1 3D structure was obtained from the RCSB PDB web server ( https://www.rcsb.org) Keap1 was then prepared to remove water molecules and ligands with BIOVIA Discovery Studio 2019.

### Molecular analysis of docking and ligand-receptor interactions

Ligands-Keap1 interactions were analysed by molecular docking using AutoDock Vina integrated in PyRx version 0.9.5. The molecular docking process is carried out using a specific docking method based on the active site of the Keap1 protein. ^17^^,^^22^ Docking results, bond positions, and amino acid residues formed between ligands-receptor were analysed using PyMol software and BIOVIA Discovery Studio 2019.

### Molecular dynamic simulation

Molecular dynamic simulations were carried out using Yet Another Scientific Artificial Reality Application (YASARA). The molecular dynamic simulation aims to compare the interaction complexes of Betaine, Choline, TEMPO, and Keap1-binding inhibitors. The parameters in the simulation correspond to the physiological conditions of the cells, namely temperature 37 °C, 1 atm, pH 7.4, and 0.9% salt content for 50 ns with autosaved every 25 ps. The simulation is run by the md_run macro program, and the results are displayed by the md_analyze and md_analyzeres programs. ^24^^–^^26^

## Results

### Phytochemical properties and antioxidant activity of *S. caseolaris* leave extract

The mass yield percentage, pH, total flavonoids, total phenols, and antioxidant activity of *S. caseolaris* mangrove leaves extracted using various solvents yielded different results. Mangrove leaves of *S. caseolaris* extracted with methanol (E) and distilled water (A) exhibited high antioxidant activity, with IC _50_ values of 63.72 ± 1.27 mg/mL and 89.29 ± 006 mg/mL respectively. The high antioxidant activity was supported by the total amount of flavonoids and total phenol of both extracts, which were higher than the other extracts. In contrast, mangrove leaves extracted with bottled drinking water (Ax, Cx, and Kx) showed low antioxidant activity. Mangrove leaves extracted with distilled water (A), Ax brand bottled mineral water (B), and methanol (E) have a pH of less than 7 ( Table 1). ^48^

**Table 1.** Phytochemical properties, antioxidant activity, and mass yield percentage of various *S. caseolaris* leaves water extract.

Sample	IC50 (mg/mL)	Flavonoid content (mgQE/g)	Phenolic content (mgGAE/g)	pH	Yield (%)
A	89.29 ± 006 ^b^	0.96 ± 0.01 ^d^	1.00± 0.06 ^b^	5.0 ± 0.01 ^a^	6.06 ± 0.05 ^b^
B	810.39 ± 10.86 ^c^	0.89 ± 007 ^c^	0.38± 0.01 ^a^	6.97± 0.01 ^c^	5.72± 0.07 ^a^
C	159.36 ±4.69 ^d^	0.55 ± 0.00 ^a^	0.41± 0.01 ^a^	7.3 ± 0.01 ^c^	5.9 ± 0.04 ^b^
D	1147 ± 1.27e	0.64 ± 0.06 ^b^	0.23 ± 0.01 ^a^	7.3 ± 0.01 ^c^	6.3 ± 0.05 ^c^
E	63.72± 1.27 ^a^	1.98 ± 0.08 ^e^	1.52 ±0.02 ^b^	6.2 ± 0.01 ^b^	7.6± 0.02 ^d^

**Figure 1.**
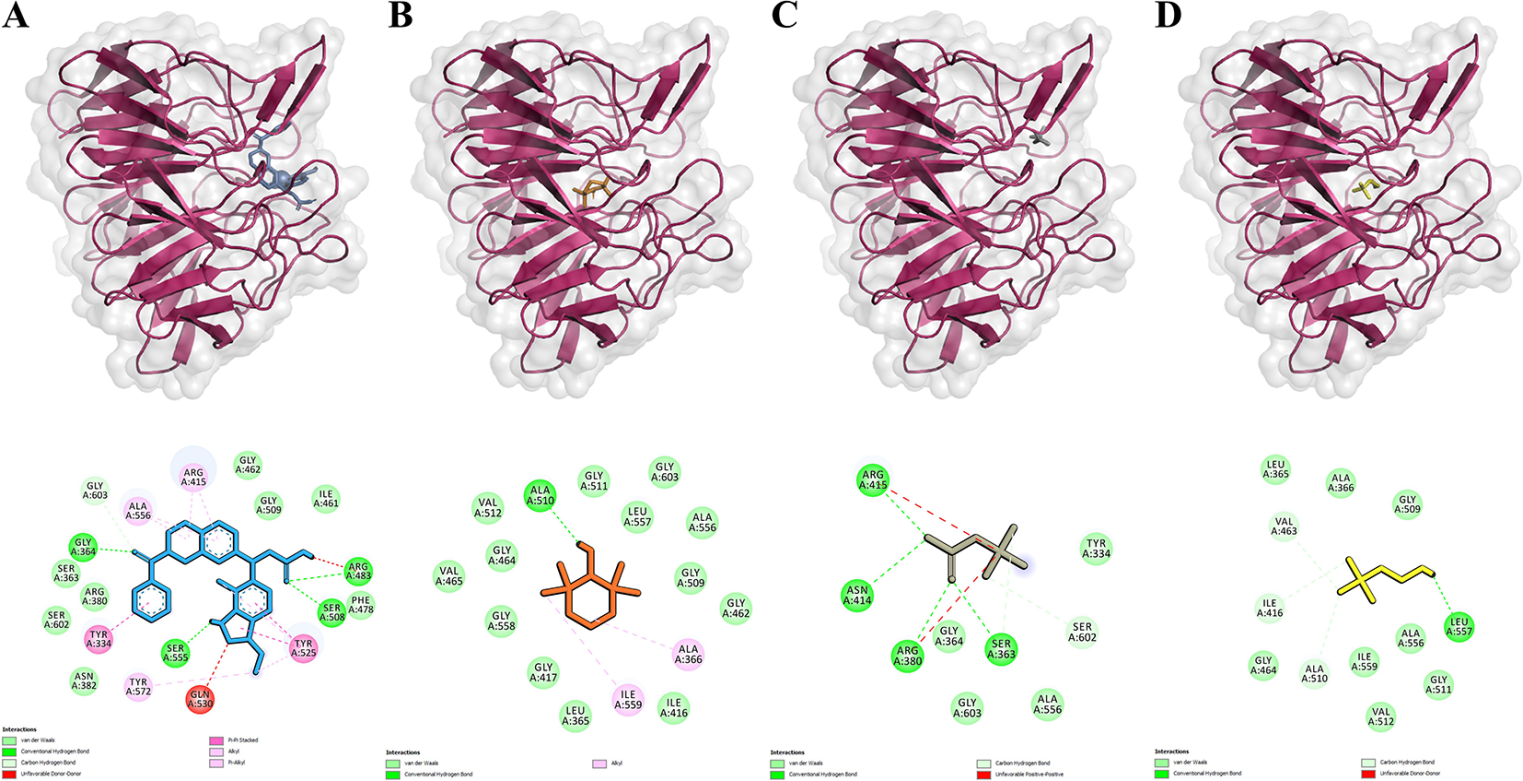
Ligands-Keap1 interactions and the amino acid residues involved. A-D. Visualization of molecular docking results between inhibitors, TEMPO, Betaine, and Choline with Keap1 protein. The top row shows each ligand's position that binds to the Keap1 protein. The bottom row shows the amino acid residues involved in the interactions of the inhibitor, TEMPO, Betaine, and Choline with the Keap1 protein. Keap1 protein is presented as a pink ribbon. The compounds are presented in the form of colored sticks, namely inhibitors (blue), TEMPO (orange), Betaine (gray), and Choline (yellow).

The content of bioactive compounds in mangrove leaf extract of *S. caseolaris* was analyzed using LC-HRMS. The analysis results revealed the presence of 16 identical active compounds in all *S. caseolaris* mangrove leaf extracts obtained with different solvents ( Table 2). The bioactive compound content in mangrove leaf extract from *S. caseolaris* was assessed through LC-HRMS. The findings from the analysis indicated the presence of 16 identical active compounds in all *S. caseolaris* mangrove leaf extracts, regardless of the solvents used (refer to Table 2). From the comprehensive selection, three compounds were identified exhibiting antioxidant activity: TEMPO, Betaine, and Choline. Figure 1 Displays the 2D and 3D representations of these substances interacting with the Keap1 protein.

**Table 2.** Bioactive compounds of various *S. caseolaris* leaves water extract using LC-HRMS.

Compound	Formula	Molecular weigh	RT (min)	A	B	C	D	E
				Area (Max)	Area (Max)	Area (Max)	Area (max)	Area (max)
Diisobutylphthalate	C16 H22 O4	278.15	18.1	1,478,572,592.33	1,848,386,564.31	1,436,638,448.14	1,433,462,396.74	607,892,729.57
2,2,6,6-Tetramethyl-1-piperidinol (TEMPO)	C9 H19 N O	157.14	12.487	624,439,633.82	1,572,283,545.52	748,454,617.62	682,355,229.32	607,892,729.57
Betaine	C5 H11 N O2	117.07	0.914	417,836,215.50	664,516,094.35	277,354,257.65	510,638,218.10	251,262,790.44
Hexamethylenetetramine	C6 H12 N4	140.11	26.437	263,891,780.96	323,365,164.64	312,130,282.75	273,062,719.37	264,426,927.99
Choline	C5 H13 N O	103.10	1.173	151,720,733.01	157,800,900.57	66,818,394.14	304,604,229.85	211,612,525.07
Bis(3,5,5-trimethylhexyl) phthalate	C26 H42 O4	418.31	0.909	91,904,530.77	87,553,704.16	166,099,696.24	-	97,131,362.57
Caprolactam	C6 H11 N O	113.08	3.603	70,653,446.85	68,967,610.79	68,991,197.52	67,857,854.99	98,754,094.35
2-[(2-chlorobenzyl)sulfanyl]-4,6-dimethylnicotinonitrile	C15 H13 Cl N2 S	326.00	0.903	70,636,702.51	74,226,085.12	119,600,241.54	-	91,828,976.68
3,5-di-tert-Butyl-4-hydroxybenzaldehyde	C15 H22 O2	234.16	17.145	59,098,869.65	69,027,475.30	58,235,830.03	48,272,305.90	56,803,228.75
Zearalenone	C18 H22 O5	300.13	18.254	57,574,586.69	-	-	-	201,277,679.34
Tributyl phosphate	C12 H27 O4 P	266.16	16.711	56,340,547.62	74,766,342.63	52,529,750.78	49,942,348.93	58,094,706.32
Bis(4-ethylbenzylidene)sorbitol	C24 H30 O6	414.20	14.809	54,768,325.03	41,035,738.19	41,575,103.25	60,527,533.49	126,160,191.57
Monobutyl phthalate	C12 H14 O4	222.09	17.132	23,908,635.26	176,996,556.64	134,104,756.25	108,363,745.74	94,078,367.66
Bis(2-ethylhexyl) phthalate	C24 H38 O4	390.27	22.766	12,616,982.48	157,896,657.47	24,131,565.18	60,358,628.37	56,794,541.13
DL-Arginine	C6 H14 N4 O2	174.11	1.177	93,941,509.47	85,395,666.15	46,382,487.63	96,900,587.14	12,458,213.51
2-[(2-chlorobenzyl)sulfanyl]-4,6-dimethylnicotinonitrile	C15 H13 Cl N2 S	326.00	16.809	70,636,702.51	74,226,085.12	119,600,241.54	-	91,828,976.68

### Ligand-Keap1 molecular interactions

This study compared the binding activities of Betaine, Choline, TEMPO, and inhibitors to the Keap1 protein. The inhibitor used is a natural inhibitor that binds to the active site of the Keap1 protein and serves as a control. Molecular docking results were visualized with PyMol and Discovery Studio to identify the binding site of Ligand-Keap1.

Molecular docking results indicated that the TEMPO-Keap1 interaction possesses a lower binding affinity value compared to the Betaine-Keap1 and Choline-Keap1 interactions, which have values of -5.5 kcal/mol, -4.2 kcal/mol, and -3.7 kcal/mol, respectively. The lower binding affinity value observed for TEMPO-Keap1 interaction suggests its strength is greater than that of the Betaine-Keap1 and Choline-Keap1 interactions. Notably, the binding affinity value of TEMPO-Keap1 is higher than that of Inhibitor-Keap1, which exhibits a binding affinity value of -11.1 kcal/mol ( Table 3).

**Table 3.** Molecular docking results and amino acid residues on Ligand-Keap1 interaction.

CID	Ligand	Binding affinity (kcal/mol)	Amino acid residues	
			Hydrogen interaction	Hydrophobic interaction
549976	TEMPO	-5.5	Ala510	Leu365, Ala366, Ile416, Gly417, **Gly462**, Gly464, Val465, **Gly509**, Gly511, Val512, **Ala556**, Leu557, Gly558, Ile559, **Gly603**
247	Betaine	-4.2	**Ser363**, **Arg380**, Asn414, **Arg415**	**Tyr334**, **Gly364**, **Arg380**, **Arg415**, **Ala556**, **Ser602**, **Gly603**
305	Choline	-3.7	Leu557	Leu365, Ala366, Ile416, Val463, Gly464, **Gly509**, Ala510, Gly511, Val512, **Ala556**, Ile559
135263934	Inhibitor	-11.1	**Gly364**, Arg483, Ser508, Ser555	**Tyr334**, **Ser363**, **Arg380**, Asn382, **Arg415**, Ile461, **Gly462**, Phe478, **Gly509**, Tyr525, Gln530, **Ala556**, Tyr572, **Ser602**, **Gly603**

The visualization results revealed that Betaine shares the same binding site as the inhibitor (control), whereas TEMPO and Choline do not exhibit a specific binding site. They are overlap with the binding of the inhibitor ( Figure 1 on the top row). The visualization process continued with the identification of the amino acid residues involved in the Ligand-Keap1 interaction. It is observed that the Betaine-Keap1 interaction shares the same amino acid residues as the inhibitor-Keap1 interaction, and these interactions involve the highest number of residues when compared to the TEMPO-Keap1 and Choline-Keap1 interactions. Specifically, Ser363, Arg380, and Arg415 were identified as three amino acid residues engaged in hydrogen interactions on Betaine-Keap1. Furthermore, the visualization results indicated that Arg380 and Arg415 in Betaine-Keap1 interaction were also involved in hydrophobic interactions. Additionally, five other amino acid residues engaged in hydrophobic interactions in the Betaine-Keap1 were Tyr334, Gly364, Ala556, Ser602, and Gly603 ( Table 3 and Figure 1 in the bottom row).

Four amino acid residues are shared between the TEMPO-Keap1 and inhibitor-Keap1 interactions, namely Gly462, Gly509, Ala556, and Gly603, all of which are involved in hydrophobic interactions. The Choline-Keap1 interaction shares the same two amino acid residues as the inhibitor-Keap1 interaction, namely Gly509 and Ala556, and these interactions are also hydrophobic in nature. The presence of identical amino acid residues among TEMPO, Betaine, Choline, and the inhibitor in the Keap1 protein suggests the possibility that these compounds may exhibit similar activity to the inhibitor (control).

The binding affinity of the ligand-receptor complex increases when amino acid residues are involved in the interaction through hydrogen bonds and hydrophobic bonds.

Although, the Betaine-Keap1 interaction involves more amino acid residues than the TEMPO-Keap1 interaction, the binding affinity value of Betaine-Keap1 is higher than that of TEMPO-Keap1. It is possible that the amino acid residues Gly462 and Gly509 play a specific role in the TEMPO-Keap1 interaction, and these particular residues are not found in the Betaine-Keap1 interaction ( Table 3 and Figure 1 in the bottom row).

It’s important to note that a higher binding affinity value in the TEMPO-Keap1 interaction does not necessarily imply that it is weaker than the inhibitor-Keap1 interaction with its lower binding affinity value.

After molecular docking, molecular dynamics (MD) simulations were conducted to assess the stability of the Ligand-Keap1 interaction complex. The parameters employed in this simulation included the Root Mean Square Deviation (RMSD) of the Ligand-Keap1 complex, RMSD of Ligand movement, Root Mean Square Fluctuation (RMSF), and the number of hydrogen bonds within the Ligand-Keap1 complex. ^22^

**Figure 2.**
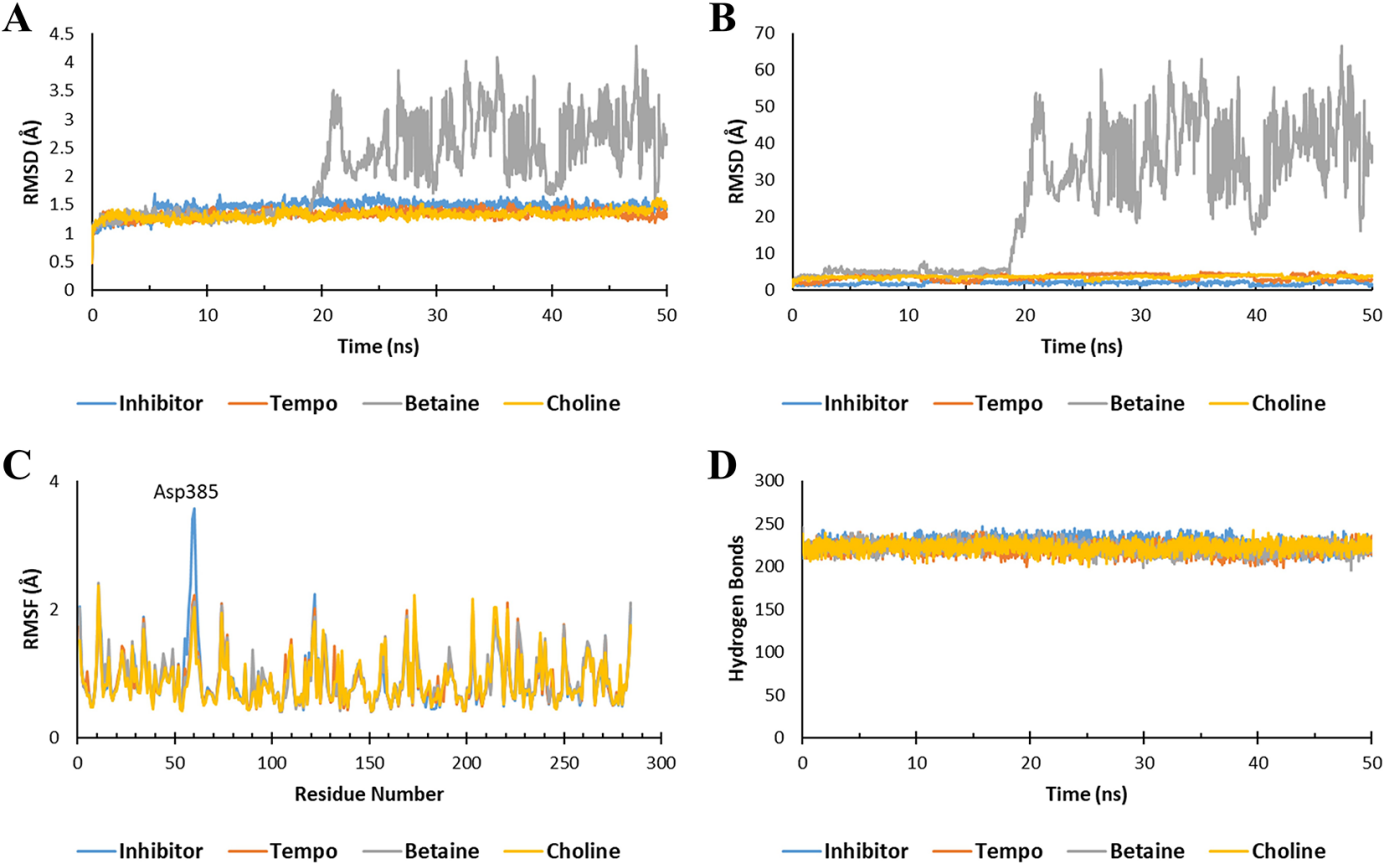
Molecular dynamic simulation. The stability of the ligand-Keap1 interaction complex during the simulation was indicated by (A) the RMSD of the ligand-Keap1 complex value, (B) the RMSD of the Ligand movement value, (C) the RMSF value, and (C) the number of hydrogen bonds.

The molecular dynamic analysis results indicated that both the TEMPO-Keap1 and Choline-Keap1 complexes exhibited similar stability to the Keap1 inhibitor throughout the simulation, as evidenced by an RMSD value of <2 Å. On the other hand, the Betaine-Keap1 complex at the beginning of the simulation up to 20 ns also showed the same stability, but after 20 ns the complex was unstable until the end of the simulation with an RMSD value of >3 Å. The RMDS Ligand movement results further supported the observation of Betaine’s instability after 20 ns, with significantly differing RMSD values when compared to TEMPO, Choline, and inhibitors ( Figures 2A and 2B). These findings align with previous studies, which reported that a stable ligand-receptor complex during the simulation typically maintain an RMSD value of 3 Å. ^27^^,^^28^

The fluctuation of the amino acid residues also serves as an indicator of the stability of the ligand-Keap1 complex. The results demonstrated that all complexes exhibited nearly identical fluctuations in amino acid residues ( Figure 2C). Previous research has indicated that a higher degree of fluctuation among amino acid residues correlates with increased instability in the e ligand-receptor complex. ^29^ In all interaction complexes, more than 200 hydrogen bonds were observed ( Figure 2D). The abundance of hydrogen bonds within these complexes indicates their stability. However, it’s important to note that the stability of the Betaine-Keap1 interaction requires further analysis using additional parameters to ensure a conclusive determination. This is because the results of the RMSD Ligand-Keap1 complex, RMSD Ligand movement, RMSF, and the number of hydrogen bonds of the Betaine-Keap1 complex exhibit variations.

## Discussion

Previous studies have indicated that mangrove leaves have very high antioxidant activity. Ethanol extract of of *S. caseolaris* mangrove leaves demonstrated high antioxidant activity, which varies with the leaves’ maturity level. ^9^^,^^30^ Other studies have also suggested that the high antioxidant activity of *S. caseolaris* mangrove leaf extract can be attributed to the presence of several active compounds, such as flavonoids and tannins. ^9^

In the present study (un-publish data), mangrove leaves of *S. caseolaris* extracted using various types of solvents have shown potential as antioxidant agents, as determined by the DPPH method. Among the extracts, *S. caseolaris* leaves extracted with methanol (control) and distilled water exhibited the highest antioxidant activity compared to other extracts, for which IC50 values were determined. This is supported by the higher flavonoid and phenolic content found in the methanol and distilled water extracts, as compared to other extracts. These results were consistent with lower levels total flavonoids and total phenols compared to treatments A and E. Based on both our research findings and prior studies, it is well-established that total flavonoids and total phenols exert a substantial influence on high antioxidant activity. ^31^^,^^32^

Mangrove leaves contain varying levels of salt depending on the salinity of their growing environment, as evidenced by leaf age, ^33^ the presence of lenticels on the leaves, ^34^ and the existence of leaf salt glands. ^35^ Mineral water samples vary in mineral content and pH levels. It is hypothesized that the interaction between the salt content in mangrove leaves and the salts from mineral water contributes to the higher IC50 values compared to those of distilled water and methanol extracts. Distilled water is more effective in dissolving polar compounds present in the leaves without being affected by the mineral ions in the mangrove leaves. Nevertheless, mineral water is considered more practical and safer as an extracting solvent.

The LC-HRMS analysis detected 16 components present in all the extracts examined., Among these, TEMPO, Betaine, and Choline were identified as active compounds known for their antioxidant properties. ^36^^,^^37^ TEMPO finds extensive use in cellular and animal model studies due to its favourable characteristics, including low molecular weight, high water solubility, and the capability to permeate cell membranes efficiently. ^38^ Betaine, a natural compound, is a glycine derivative with three additional methyl groups, as depicted in Figure 1 in both 2D and 3D forms. It is widely found in plants, animals, and microorganisms, including beets, spinach, wheat bran, wheat germ, and aquatic invertebrates. Betaine can be produced endogenously through choline metabolism or obtained exogenously through dietary intake, exhibiting similar bioavailability whether consumed as a supplement or through food, ultimately metabolizing to dimethylglycine and sarcosine in kidney and liver cells’ mitochondria. ^39^

Choline, a vital nutrient, plays a fundamental role in diverse biological functions such as the synthesis of structural lipoproteins and membrane lipids, neurotransmitters, and the maintenance of brain function. Betaine, derived from choline oxidation, is crucial for the formation of essential amino acids like methionine. Dimethylglycine (DMG), a betaine metabolite, serves as a vital source of glycine and methyl groups for biochemical reactions. Additionally, trimethylamine N-oxide (TMAO) is metabolized in the liver from trimethylamine (TMA), primarily generated from dietary choline and betaine, and emerges as a risk factor in various diseases, including renal disease, cardiovascular disease, colorectal cancer, type II diabetes, and neurological disorders. This highlights the multifaceted role of choline metabolism and its metabolites in health and disease. ^40^

This research showed that molecular docking and molecular dynamics simulations assessed the differential binding affinities and stability of Ligand-Keap1 interactions involving Betaine, Choline, and TEMPO. This is because in molecular docking simulations consider multiple parameters to determine the strength or weakness of the ligand-receptor interaction. These parameters include the binding affinity value, hydrogen interaction, and hydrophobic interactions formed on amino acid residues between the ligand-receptor, and molecular dynamic results. ^41^ The binding affinity of the ligand-receptor complex increases when amino acid residues are involved in the interaction through hydrogen bonds and hydrophobic bonds. Furthermore, it is well-known that hydrophobic interactions play a significant role in stabilizing the ligand-protein bond and contribute to enhancing the ligand’s affinity for the protein. ^42^^,^^43^

Molecular docking is recognized as one of the vital methods in molecular simulation. This method is based on the recognition process involving spatial matching and intermolecular energy matching between two or more molecules. Molecular docking is an effective and efficient approach that helps reduce costs and time in studying the mechanism of compound activity. ^44^

TEMPO, Betaine, and Choline are recognized as active compounds with antioxidant properties. ^45^ Previous studies have demonstrated that Choline significantly enhances the activity of glutathione S-transferase, superoxide dismutase, catalase, and glutathione peroxidase Other research has indicated that TEMPO, when conjugated with specific compounds, can effectively reduce oxidative stress caused by hydrogen peroxide. ^36^ Betaine, functioning as an antioxidant agent, has been shown to significantly decrease malondialdehyde (MDA) levels while increasing the activity of superoxide dismutase (SOD) and glutathione peroxidase (GPx). ^37^^,^^46^ Based on the findings of these studies, the present research employed TEMPO, Choline, and Betaine as potential candidates for inhibiting the Keap1 protein, thereby enhancing antioxidant activity in the body. The result providing valuable insights into the potential activities of Betaine, Choline, and TEMPO as compared to the control inhibitor. The results showed that the ability of Betaine, Choline, and TEMPO to inhibit the Keap1 protein, which acts as a Nrf2 inhibitor using a molecular docking approach.

Oxidative stress is an imbalance between free radicals-antioxidants in the body, resulting in damage and contributing to various diseases, such as cancer, diabetes, neurodegenerative diseases, and aging. ^17^ Nuclear factor erythroid 2-related factor 2 (Nrf2) is a protein that plays an important role in preventing oxidative stress. Nrf2 is the primary regulator of the cellular stress response, triggering the expression of antioxidant enzymes that protect cells from oxidative stress induced. ^22^^,^^47^ Under normal circumstances, Nrf2 is bound by the endogenous inhibitor Kelch-like ECH-associated protein 1 (Keap1) in the cytosol. The Keap1 protein contains cysteine residues that can bind to ROS when oxidative stress occurs, leading to disruption of the Nfr2-Keap1 bond. This disruption results in phosphorylation and translocation of Nrf2 into the cell nucleus. Translocated Nrf2 then binds to a DNA regulatory region known as the ARE (Antioxidant Response Element). The Nrf2-ARE binding activates the transcription of genes responsible for encoding antioxidant enzymes, including superoxide dismutase, catalase, glutathione peroxidase, thioredoxin reductase, glutamate cysteine ligase, and others. ^18^^–^^21^

In conclusion, the results revealed the presence of the TEMPO, Choline, and Betaine compounds with antioxidant potential in all extracts. The antioxidant potential of these three compounds was further confirmed through by the results of molecular docking, indicating their ability to inhibit the Keap1 protein - endogenous Nrf2 inhibitor, and the enhance the activity of enzymes involved in antioxidant processes. Further research is necessary to validate the potential of *S. caseolaris* leaves as an antioxidant agent, with the aim of utilizing them as bioactive food ingredients and increasing antioxidant intake for consumers.

## Data Availability

Figshare: The potential of Sonneratia caseolaris mangrove leaves extract as a bioactive food ingredient using various water extract, https://doi.org/10.6084/m9.figshare.25100123.v1. ^48^ Data are available under the terms of the Creative Commons Attribution 4.0 International license (CC-BY 4.0).
