# Supplementary material for: The potential of  Sonneratia caseolaris mangrove leaves extract as a bioactive food ingredient using various water extract
Source: F1000Res. 2025 Jan 3;13:249. Originally published 2024 Apr 4. [Version 4] doi: 10.12688/f1000research.143708.4 (PMC11754952; doi:10.12688/f1000research.143708.4)
Supplement: Peer review history

# Reviewer report: Reviewer response for version 4

**DOI:** 10.5256/f1000research.176243.r358921

**Published:** 2025-01-28

**Authors:** Made Dharmesti Wijaya

**Affiliations:** 1 Fakultas Kedokteran dan Ilmu Kesehatan Universitas Warmadewa, Denpasar, Indonesia

I have carefully read this manuscript along with the previous review reports. The research is highly interesting and the study's aims have been successfully achieved using detailed and thorough methods. Below are a few additional points for consideration:

 1. The background in the abstract provides valuable context on the traditional use and bioactivity of *S. caseolaris* leaves, but it does not explicitly link these aspects to the potential of the extract as a bioactive food ingredient, as stated in the title. To strengthen the abstract, I recommend including a brief explanation of how the study’s assessment of antioxidant activity relates to the development or application of *S. caseolaris* leaves as a bioactive food ingredient. This would create a clearer connection between the background, the study’s aims, and the title.

 2. In the abstract, sentence "The resulting extract was evaluated for antioxidant activity by the 2,2-diphenyl-1-picrylhydrazyl radical scavenging activity (DPPH) techniques." in the Background part should be moved into Methods part.

 3. In the sampling method, the authors stated that the leaves sample were collected from **a mangrove plant**, but in the following sentence the authors wrote "...collected from the third to the fifth leaf, counted from the top of **each plant**". So I am wondering, was the sample taken from 1 plant or several plants? If the samples were taken from several plants, please explain further how many plants and whether they were taken from different locations? Or do you mean "from the top of each branch"? 

 4. In the statistical analysis, the method described is missing mention of the homogeneity of variance test, which is a key assumption of ANOVA alongside the normality test. 

 5. Paragraph 3 of the Discussion lacks a clear connection. 

**Sentence 3** ("It is hypothesized..."), introduces a hypothesis about the interaction between salt content and mineral water, which might influence the higher IC50 values. However, it does not explicitly connect to the solvents' role in extracting polar compounds or explain why this interaction matters in the context of the discussion.

**Sentence 4** ("Distilled water is..."), shifts to discussing distilled water's effectiveness in dissolving polar compounds. However, it does not explain how this is related to the hypothesis mentioned earlier or why polar compounds are important in the context of antioxidant activity.

**Sentence 5** ("Nevertheless, mineral water is..."), introduces the idea of practicality and safety, which feels disconnected from the previous discussion about solvent effectiveness and salt interactions. There is no explanation of how practicality and safety balance against the IC50 results or compound extraction efficiency.

 6. This article has mostly cited current literatures published above 2020. Although some of the literatures are 6-10 years old, I do think that they are still relevant.

Is the work clearly and accurately presented and does it cite the current literature?

Yes

If applicable, is the statistical analysis and its interpretation appropriate?

Partly

Are all the source data underlying the results available to ensure full reproducibility?

Yes

Is the study design appropriate and is the work technically sound?

Yes

Are the conclusions drawn adequately supported by the results?

Yes

Are sufficient details of methods and analysis provided to allow replication by others?

Yes

Reviewer Expertise:

pharmacology, herbal medicines, bioprospecting. medical biotechnology

I confirm that I have read this submission and believe that I have an appropriate level of expertise to confirm that it is of an acceptable scientific standard, however I have significant reservations, as outlined above.

# Reviewer report: Reviewer response for version 4

**DOI:** 10.5256/f1000research.176243.r355697

**Published:** 2025-01-22

**Authors:** Hai-Lei Zheng

**Affiliations:** 1 Xiamen University, Xiamen, Fujian, China

I accepted the author's explain for the comment 19, and no more comments anymore on this MS.

Is the work clearly and accurately presented and does it cite the current literature?

Partly

If applicable, is the statistical analysis and its interpretation appropriate?

Yes

Are all the source data underlying the results available to ensure full reproducibility?

Partly

Is the study design appropriate and is the work technically sound?

Yes

Are the conclusions drawn adequately supported by the results?

Yes

Are sufficient details of methods and analysis provided to allow replication by others?

Yes

Reviewer Expertise:

NA

I confirm that I have read this submission and believe that I have an appropriate level of expertise to confirm that it is of an acceptable scientific standard.

# Reviewer report: Reviewer response for version 3

**DOI:** 10.5256/f1000research.169736.r347083

**Published:** 2024-12-16

**Authors:** Hai-Lei Zheng

**Affiliations:** 1 Xiamen University, Xiamen, Fujian, China

Review comments

 I am not the reviewer for this manuscript from version1 to version3. I have carefully read the previous reviewing results. Basically, I agree with the previous reviewers’ comments. After reading the manuscript, however, I have another comments on the MS.

 This MS investigated various water extracts from the leaves of mangrove plant *Sonneratia caseolaris*. Using phytochemical methods, LC-HRMS, and bioinformatics approaches, the authors claimed that the extract by distilled water exhibited the highest antioxidant activity compared to other three brands of bottled drinking water. Three compounds including Tempo, choline, and betaine in all *S. caseolaris* leaf extracts were identified, and their interactions with the putative target protein Keap1 were analysed. The results provide a possible utilization of *S. caseolaris* leaf extracts as an important antioxidant in food industry. Although the authors obtained some useful data, performed appropriate analyses and revised several rounds, the MS have large space to improve the MS. Here are some points.

1. As last reviewer questioned why did authors use 3 types of bottled drinking water, the authors should provide a simple explanation in main text in the MS rather than just explained in the response letter.
2. Here, I have another question why the extract by distilled water exhibited the highest antioxidant activity compared to other three brands of bottled drinking water. Do you have any explanation or discussion on this results? The authors should provide some suggestions on the use of distilled water or bottled drinking water on the economically and efficiently basis.
3. In the whole MS, the usage of Tempo, Choline, and Betaine should be revised. Here, Tempo is 2,2,6,6-tetramethyl-1-piperidinol (see Table 2), the authors should provide the full name in the text when it appeared in first time. Because this is an abbreviation, you can use all capital letters like shown in Table 2, rather than Tempo. In the other hand, Choline, and Betaine is the common chemicals, it is not necessary to use the capital letter for the first letter of those two words.
4. In the Abstract, Conclusions subsection, why there are two “an” before and after the word of effective?
5. Next line, how to understand the simultaneous use of “elucidate confirm”？
6. Same line, Sonneratia caseolaris should be italic, and the name of genus can be shortened as *S*. except the first appear of *Sonneratia caseolaris*. Please check whole MS.
7. The last sentence of this subsection, it is hard to read “S. caseolaris, in order to utilize them as active components in food and enhance antioxidant consumption among consumers.” You should reword this sentence.
8. Keyword, mangrove leaves can be revised as mangrove, leaf extracts
9. In Intro, 2 ^nd^ paragraph, “S. caseolaris, commonly referred to as red pidada, is extensively employed by the Indonesian people due to its wide range of uses.” need a reference. Similarly, the sentence 1 to 3 of paragraph 3 need their own references respectively.
10. Same paragraph, this statement “Therefore, in this study, it is anticipated that the antioxidants found in *S. caseolaris* will be able to bind to Keap1, allowing Nfr2 to function effectively within the cell” is not appropriate, it’s too arbitrary.
11. Next paragraph, “Previous research has shown that S. caseolaris leaves extracted with organic solvents such as ethanol and methanol exhibit high antioxidant activity.” citation reference??
12. Please provide the data of latitude and longitude  for Ujung Pangkah Gresik, East Java, Indonesia.
13. In Sample extraction section, “The bottle drinking water (B, C, and D) was sourced from the Indonesian market. The extraction application of Sonneratia caseolaris in society will be easier to perform using bottled drinking water. Bottled drinking water is a type of mineral commonly used in Indonesia. ” delete 2 ^nd^ sentence, combine 1 ^st^ and 3 ^rd^ sebtences. Please provide the citing reference for “based on previous research findings.”
14. Microplate reader, M àm
15. Acetonitrileà acetonitrile
16. Add “.” after autosampler.
17. Molecular dynamic simulation section, give full name for MD
18. Result section, what do you mean “leave extract”？
19. First subtitle of Results section and the Table 1 title, the authors all mentioned the word of antioxidant activity, as the authors mentioned the method to measure the antioxidant activity in Methods. However, in this subsection, there are no data presented for antioxidant activity whatever in Table 1 and main text, although there is IC50. I think it’s different  between IC50 and antioxidant activity.
20. Page 7, the 2 ^nd^ and 3 ^rd^ paragraph is the discussion, should move to discussion section. In the results section, authors only present their own data, no citation reference, no other data produced from other studies.
21. In the 2 ^nd^ paragraph of Ligand-Keap1 molecular interactions section, delete “Molecular docking results indicated that”, it repeated with last sentence in last paragh.
22. Too many “However”
23. Something missed in the beginning of 3 ^rd^ sentence of 1 ^st^ parag. in Discussion section.
24. Missing “.” and citation reference after “Previous studies have demonstrated that Choline significantly enhances the activity of glutathione S-transferase, superoxide dismutase, catalase, and glutathione peroxidase”
25. Missing page information in reference number 5,9,10,16,17. The latin name should be italic in reference number 11, 14.

Is the work clearly and accurately presented and does it cite the current literature?

Partly

If applicable, is the statistical analysis and its interpretation appropriate?

Yes

Are all the source data underlying the results available to ensure full reproducibility?

Partly

Is the study design appropriate and is the work technically sound?

Yes

Are the conclusions drawn adequately supported by the results?

Yes

Are sufficient details of methods and analysis provided to allow replication by others?

Yes

Reviewer Expertise:

Mangrove ecological physiology, Plant physiology and biochemistry

I confirm that I have read this submission and believe that I have an appropriate level of expertise to confirm that it is of an acceptable scientific standard, however I have significant reservations, as outlined above.

# Response

**Published:** 2024-12-20

**Authors:** Hartati Kartikaningsih

**Affiliations:** Postgraduate School, Universitas Brawijaya, Malang, East Java, Indonesia

Thanks for the review

 Due to the extensive feedback received, I have corrected most of the reviews, and you can see these changes once the updated version is published. As for review number 19, since it involves a more complex issue, I will address it in this response

 In the methodology, we have included the antioxidant testing procedures and the calculation of IC50 values. Antioxidant activity refers to the ability of a substance to act as an antioxidant, specifically by mitigating potential damage to biological systems caused by free radicals. Thus, the DPPH assay was employed to assess the efficacy of the test material in neutralizing these radicals.

 The IC50 value represents the concentration of a substance required to inhibit 50% of a specified chemical process under defined conditions. In the context of antioxidants, IC50 can be interpreted as the concentration needed to reduce free radical activity by 50%. A lower IC50 value indicates a greater antioxidant potential.

 In general, antioxidant activity provides a measure of a substance’s ability to function as an antioxidant, while the IC50 value reflects its efficiency in inhibiting free radicals.

 Researchers who used antioxidant activity expressed as IC50 include Wonggo et al. (2024), Ibrahim et al. (2022), Mitra et al. (2023), and Sari et al. (2024). However, there are also studies that measure antioxidant activity expressed as equivalents to Trolox (TEAC) in µmol/g, as conducted by Hinokideni et al. (2022)

1. **Wonggo, D., Anwar, C., Dotulong, V., Reo, A., Taher, N., Syahputra, R. A., Nurkolis, F., Tallei, T. E., Kim, B., & Tsopmo, A. (2024).** Subcritical water extraction of mangrove fruit extract ( *Sonneratia alba*) and its antioxidant activity: Network pharmacology and molecular connectivity studies. *Journal of Agriculture and Food Research, 18*, 101334. https://doi.org/10.1016/j.jafr.2023.101334
2. **Ibrahim, H. A. H., Abdel-Latif, H. H., & Zaghloul, E. H. (2022).** Phytochemical composition of *Avicennia marina* leaf extract: Its antioxidant, antimicrobial potentials, and inhibitory properties on *Pseudomonas fluorescens* biofilm. *Egyptian Journal of Aquatic Research, 48*(1), 29–35. https://doi.org/10.1016/j.ejar.2021.12.003
3. **Mitra, S., Naskar, N., Lahiri, S., & Chaudhuri, P. (2023).** A study on phytochemical profiling of *Avicennia marina* mangrove leaves collected from Indian Sundarbans. *Sustainable Chemistry for the Environment, 4*, 100041. https://doi.org/10.1016/j.sce.2023.100041
4. **Sari, N. I., Nurkolis, F., & Tallei, T. E. (2024).** Valorization of mangrove leaves *Sonneratia alba* based on leaf age as functional salt preparation. *Kuwait Journal of Science, 51*, 100073. https://doi.org/10.1016/j.kjs.2023.06.003
5. **Hinokideni, K., Aoki, R., Inoue, T., Irie, M., & Nakanishi, Y. (2022).** Usability of mangrove plant leaves as tea materials: A comparison study on phenolic content and antioxidant capacities with commercial teas. *Biocatalysis and Agricultural Biotechnology, 41*, 102307. https://doi.org/10.1016/j.bcab.2021.102307

# Reviewer report: Reviewer response for version 3

**DOI:** 10.5256/f1000research.169736.r316530

**Published:** 2024-09-19

**Authors:** Neni Anggraeni

**Affiliations:** 1 University of Tsukuba, Ibaraki, Japan

This manuscript has meet the requirement for indexing.

Is the work clearly and accurately presented and does it cite the current literature?

Partly

If applicable, is the statistical analysis and its interpretation appropriate?

Partly

Are all the source data underlying the results available to ensure full reproducibility?

Partly

Is the study design appropriate and is the work technically sound?

Yes

Are the conclusions drawn adequately supported by the results?

Partly

Are sufficient details of methods and analysis provided to allow replication by others?

No

Reviewer Expertise:

herbal medicines, metabolism, stem cell biology and animal modelling

I confirm that I have read this submission and believe that I have an appropriate level of expertise to confirm that it is of an acceptable scientific standard.

# Reviewer report: Reviewer response for version 2

**DOI:** 10.5256/f1000research.166667.r283621

**Published:** 2024-06-26

**Authors:** Neni Anggraeni

**Affiliations:** 1 University of Tsukuba, Ibaraki, Japan

The revised article meets all the criteria regarding scientific matters. This article will provide knowledge through bioinformatics tool to predict the active site of some compound that useful to inhibit ROS, and it's very important before doing some in vitro or in vivo experiments. 

 1. About bottled drinking water. I do agree with you that this water is easy to get but why did you need to use 3 types of bottled drinking water? What is the difference between those 3 bottled drinking water? 

 2. About the Duncan test. The Duncan test is a post-hoc test. Before using the post-hoc test (Duncan test) what kind of test did you use to get the p-value for the whole group? please state in the statistical section

Is the work clearly and accurately presented and does it cite the current literature?

Partly

If applicable, is the statistical analysis and its interpretation appropriate?

Partly

Are all the source data underlying the results available to ensure full reproducibility?

Partly

Is the study design appropriate and is the work technically sound?

Yes

Are the conclusions drawn adequately supported by the results?

Partly

Are sufficient details of methods and analysis provided to allow replication by others?

No

Reviewer Expertise:

herbal medicines, metabolism, stem cell biology and animal modelling

I confirm that I have read this submission and believe that I have an appropriate level of expertise to confirm that it is of an acceptable scientific standard, however I have significant reservations, as outlined above.

# Response

**Published:** 2024-07-23

**Authors:** Hartati Kartikaningsih

**Affiliations:** Postgraduate School, Universitas Brawijaya, Malang, East Java, Indonesia

Title: The potential of *Sonneratia caseolaris* mangrove leaves extract as a bioactive food ingredient using various water extract

**Dear Reviewer,**

 Thank you very much for the opportunity to revise our manuscript. Thank you very much for all your valuable comments. The detailed responses are described below:

**REVIEWER COMMENT -**

 1. About bottled drinking water. I do agree with you that this water is easy to get but why did you need to use 3 types of bottled drinking water? What is the difference between those 3 bottled drinking water? 

**Author Response**:

 The difference between used 3 bottled drinking water is described below:

**Water Sample Code** **(B): **The water specified by this code originates from the Jonggol mountains in Bogor, Indonesia, and also from the base of Mount Salak in Bogor, West Java. The environmental attributes of this water source include a pH of approximately 7.2 and an oxygen concentration of 10.88 ppm. It is rich in essential minerals such as Mg, Ca, Na, and Se, and has received certification for its suitability for human consumption. To prevent the presence of microorganism contaminants that pose risks to human health, the water production process incorporates an osmosis backup system in its filtration mechanism.

**Water Sample Code** **(C): **The specifications for the drinking water provided in this sample code pertain to the various stages involved in a filtration system that employs high pressure filtration techniques. The Total Dissolved Solids (TDS) content measures less than 10 ppm, thereby indicating the successful removal of unnecessary organic minerals through the utilization of a nano filter, resulting in the attainment of water characterized as pure. This water variant exhibits a slightly astringent flavor profile, possesses a pH level of 7.3, lacks chlorine and sodium fluoride, and showcases an oxygen content of 80 parts per million.

**Water Sample Code** **(D): **This water condition refers to a variety of water utilized for consumption purposes without the necessary distribution authorization. Referred to as alkaline water, its pH level can reach up to 9.5; however, the specimen analysed in this research exhibited a pH of 7. The process of water filtration in question involves the utilization of water distillation machinery.

**REVIEWER COMMENT -**

 2. About the Duncan test. The Duncan test is a post-hoc test. Before using the post-hoc test (Duncan test) what kind of test did you use to get the p-value for the whole group? please state in the statistical section.

**Author Response**:

 Test used use to get the p-value for the whole group are Tests of Normality and ANOVA test. The Normality Test and ANOVA test will state in the manuscript in the statistical section.

# Reviewer report: Reviewer response for version 1

**DOI:** 10.5256/f1000research.157397.r264930

**Published:** 2024-04-29

**Authors:** Neni Anggraeni

**Affiliations:** 1 University of Tsukuba, Ibaraki, Japan

The author constructs an exciting experiment, using the result of bioinformatics we can predict not only the antioxidant activity but also the binding site of the molecules for further study, before conducting in-vitro and in-vivo.

 However, I have the following concern that I hope the author can explain:

1. Please explain why you need to use various types of bottled drinking water.
2. In the introduction, it is better to elaborate on the third and 4th paragraphs to make it more engaging. Is it correct based on my understanding, that the antioxidant will bind with Keap1 so the Nfr2 will function normally? Please reconstruct those paragraphs.
3. Plant extraction especially mangrove leaves has been widely explored nowadays, so it’s not difficult to find recent citations. Some citations published more than ten years ago that are used in this paper, it’s better to replace the citation with the latest one.
4. In the method, the author did not mention the weight of the leaves and the dry leaves and why you chose the leaves from the third to the fifth leaf. 
5. Some sentences will cause misunderstanding, such as whether the author used the dry extract or fractionation to analyze the total phenol and flavonoids. In that section, you used 60 μL and 1 ml samples respectively, but you did not mention the dilution, using what kind of solution you diluted the dry extract.
6. The author did not mention the statistical test they used to get the p-value.
7. “Among these, Tempo, Betaine, and Choline were identified as active compounds known for their antioxidant properties” results section, 2nd paragraph line 5. If this statement is from other papers please put the citation. However, it’s better to add biological function analysis of those compounds regarding their antioxidant properties using analysis tools (e.g. PASS online or others) to make this article more enriched.
8. The aim of this study was to evaluate the effectiveness of the bioactive compounds in *S. caseolaris* leaf extract in inhibiting the Keap1 protein. However, I didn’t get the answer about the effectiveness of those compounds compared to the inhibitor. 

Is the work clearly and accurately presented and does it cite the current literature?

Partly

If applicable, is the statistical analysis and its interpretation appropriate?

Partly

Are all the source data underlying the results available to ensure full reproducibility?

Partly

Is the study design appropriate and is the work technically sound?

Yes

Are the conclusions drawn adequately supported by the results?

Partly

Are sufficient details of methods and analysis provided to allow replication by others?

No

Reviewer Expertise:

herbal medicines, metabolism, stem cell biology and animal modelling

I confirm that I have read this submission and believe that I have an appropriate level of expertise to confirm that it is of an acceptable scientific standard, however I have significant reservations, as outlined above.

# Response

**Published:** 2024-05-02

**Authors:** Hartati Kartikaningsih

**Affiliations:** Postgraduate School, Universitas Brawijaya, Malang, East Java, Indonesia

Dear Dr. Neni Anggraeni,

 Subject: Response to Review of Manuscript

 We would like to express our sincere gratitude for the thoughtful and constructive feedback provided on our manuscript titled "The potential of *Sonneratia caseolaris* mangrove leaves extract as a bioactive food ingredient using various water extract". Your insightful comments have significantly contributed to improving the quality and clarity of our work.

 We have carefully considered each of your suggestions and addressed them as follows:

**1. Reviewer's Comment:**

 Please explain why you need to use various types of bottled drinking water.

*Author response:*

 The extraction application of *Sonneratia caseolaris* in society will be easier to perform using bottled drinking water. Bottled drinking water is a type of mineral commonly used in Indonesia

**2. Reviewer's Comment:**

 In the introduction, it is better to elaborate on the third and 4th paragraphs to make it more engaging. Is it correct based on my understanding, that the antioxidant will bind with Keap1 so the Nfr2 will function normally? Please reconstruct those paragraphs.

*Author response:*

 Thank for your suggestion, we are going to elaborated of paragraphs 3 and 4.

 Keap1 and Nfr2 lead to ubiquitination, resulting in the mimicry of Nfr2 degradation. The degradation of Nfr2 diminishes the regulation of antioxidant response elements when oxidative stress occurs. Therefore, in this study, it is anticipated that the antioxidants found in *S. caseolaris* will be able to bind to Keap1, allowing Nfr2 to function effectively within the cell. The effectiveness of *S. caseolaris* antioxidants in binding to Keap1 is crucial for maintaining Nfr2 functionality. By preventing the degradation of Nfr2, these antioxidants ensure that the regulatory mechanisms for antioxidant response remain intact even under conditions of oxidative stress. This mechanism is vital for cellular defence against oxidative damage and underscores the potential therapeutic significance of *S. caseolaris* in combating oxidative stress-related disorders.

**3. Reviewer's Comment:**

 Plant extraction especially mangrove leaves has been widely explored nowadays, so it’s not difficult to find recent citations. Some citations published more than ten years ago that are used in this paper, it’s better to replace the citation with the latest one.

*Author response:*

 Thanks for your suggestion. Here's the revised paragraph with the old reference highlighted in green

 References

1. Sindhu S, Hiroshi T, Rikuo R: Community-based portable reefs to promote mangrove vegetation growth: bridging between ecological and engineering principles. Int. J. Environ. Res. Public Health. 2021 12; 590.
2. Nur SH, Hilmi E: The correlation between mangrove ecosystem with shoreline change in Indramayu coast. IOP Conf. Ser. Earth Environ. Sci. 2021; 819: 012015.
3. Eddy S, Rasyid RM, Iskandar I, et al.: Community-Based Mangrove Forests Conservation for Sustainable Fisheries. J. Silvikultur Trop. 2016; 07: 42–47.
4. Umilia E, Asbar: Formulation of mangrove ecosystem management model based on Eco-minawisata in the Coastal Sinjai, South Sulawesi. Procedia. Soc. Behav. Sci. 2016; 227: 704–711.
5. Alongi DM: Impact of global change on nutrient dynamics in Mangrove Forests. Forests. 2018; 9.
6. Alongi DM: Impacts of climate change on blue carbon stocks and fluxes in mangrove forests. Forests. 2022; 13: 149.
7. Sarhan M, Tawfik R: The economic valuation of mangrove forest ecosystem services: Implications for protected area conservation. Georg. Wright Forum. 2018; 35: 341.
8. Hamilton SE, Casey D: Creation of a high spatio-temporal resolution global database of continuous mangrove forest cover for the 21st century (CGMFC-21). Glob. Ecol. Biogeogr. 2016; 25: 729–738.
9. Audah KA, Ettin J, Darmadi J, et al.: Indonesian mangrove Sonneratia caseolaris leaves ethanol extract is a potential super antioxidant and anti Methicillin-Resistant Staphylococcus aureus Drug. Molecules. 2022; 27.
10. Soeprobowati TR, Anggoro S, Puryono S, et al.: Species composition and distribution in the mangrove ecosystem in the city of Bengkulu, Indonesia. Water (Switzerland). 2022; 14.
11. Sukrit T, Ekaphan K, Prasart K: Comparative anatomy and salt management of Sonneratio caseolaris (L.) Engl. (Lythracear) gron in saltwater and freshwater. PeerJ. 2021; 9: e10962.
12. Takarina ND:  Mangrove root diversity and structure (cone, pencil, prop) effectiveness in accumulating Cu and Zn in sediments and water in River Blanakan. IOP Conf. Series: Earth Env. Sci 550: 012009.
13. Pratiwi DY: Antimicrobial potential of Sonneratia alba and Sonneratia caseolaris against shrimp pathogens. Asian J. Fish Aquat. Res. 2021; 12: 7–14.
14. Tran TK, Ha PTT, Henry RJ, et al: Polyphenol contents, Gas Chromatography-Mass Spectrometry (GC-MS) and antibacterial activity of methanol extract and fractions of *Sonneratia Caseolaris* fruits from Ben Tre Province in Vietnam. J. Microbiol. Biotechnol. 2024 28:94-102.
15. Bokshi B, Zilani MNH, Hossain H, et al.: Bioactivities of Sonneratia caseolaris (Linn) leaf and stem using different solvent systems. Biomed. J. Sci. Tech. Res. 2020; 31: 24578–24582.
16. Dev S, Acharyya RN, Akter S, et al.: Toxicological screening and evaluation of anti-allergic and anti-hyperglycemic potential of Sonneratia caseolaris (L.) Engl. fruits. Clin. Phytoscience. 2021; 7.
17. Zhang Z, Shang Y, Li S, et al.: Molecular docking revealed the potential anti-oxidative stress mechanism of the walnut polypeptide on HT22 Cells. Foods. 2023; 12.
18. Cui W, Min X, Xu X, et al.: Role of Nuclear Factor Erythroid 2-Related Factor 2 in diabetic nephropathy. J. Diabetes Res. 2017; 2017: 1–14.
19. Hayes JD, Dinkova-Kostova AT: The Nrf2 regulatory network provides an interface between redox and intermediary metabolism. Trends Biochem. Sci. 2014; 39: 199–218.
20. Huerta C, Jiang X, Trevino I, et al.: Characterization of novel small-molecule NRF2 activators: Structural and biochemical validation of stereospecific KEAP1 binding. Biochim Biophys. Acta - Gen. Subj. 2016; 1860: 2537–2552.
21. Takeaki S, Tatsuhide T, Hiroaki O, et al: Propofol induces nuclear localization of Nrf2 under conditions of oxidative stress in cardiac H9c2 cells. 2018. PLoS ONE; 13: e0196191.
22. Ma B, Lucas B, Capacci A, et al.: Design, synthesis and identification of novel, orally bioavailable non-covalent Nrf2 activators. Bioorg. Med. Chem. Lett. 2020; 30: 126852.
23. Sembiring EN, Elya B, Sauriasari R: Phytochemical screening, total flavonoid and total phenolic content and antioxidant activity of different parts of Caesalpinia bonduc (L.) Roxb. Pharmacogn. J. 2018; 10: 123–127.
24. Bagheri M, Mohammad HF: Fluorescence spectroscopy, molecular docking and molecular dynamic simulation studies of HSA-Aflatoxin B1 and G1 interactions. J. Lumin. 2018; 202: 345–353.
25. Deeba F, Malik MZ, Naqvi IH, et al.: Potential entry inhibitors of the envelope protein (E2) of Chikungunya virus: in silico structural modeling, docking and molecular dynamic studies. Virus Disease. 2017; 28: 39–49.
26. Krieger E, Vriend G: New ways to boost molecular dynamics simulations. J. Comput. Chem. 2015; 36: 996–1007.
27. Januarti IB, Hudan T, Sulistyaningsih: The correlation of total flavonoid and total phenolic. Jurnal Farmasi Sains Dan Komunitas. 2019; 16(2): 96–103.
28. Muflihah YM, Gollavelli G, Ling YC: Correlation study of antioxidant activity with phenolic and flavonoid compounds in 12 Indonesian indigenous herbs. Antioxidants. 2021; 10: 1530.
29. Chen D, Oezguen N, Urvil P, et al.: Regulation of protein-ligand binding affinity by hydrogen bond pairing. Sci. Adv. 2016; 2: e1501240.
30. Jakaria S, Akib MK, Imrul S, Mihammad AH: Improving the binding affinity and interaction of 5-Pentyl-2-Phenoxyphenol against Mycobacterium Enoyl ACP reductase by computational approach. Informatic in Med. Unlocked. 2021;23:100528.
31. Pantsar T, Poso A: Binding affinity via docking: Fact and fiction. Molecules. 2018; 23: 1DUMMY.
32. Martínez L: Automatic identification of mobile and rigid substructures in molecular dynamics simulations and fractional structural fluctuation analysis. PLoS One. 2015; 10: 1–10.
33. Wargasetia TL, Ratnawati H, Widodo N, et al.: Bioinformatics study of sea cucumber peptides as antibreast cancer through inhibiting the activity of overexpressed protein (EGFR, PI3K, AKT1, and CDK4). Cancer Inform. 2021; 20: 117693512110318.
34. Azminah A, Radji M, Mun’Im A, et al.: In silico study of sirt1 activators using a molecular dynamic approach. Int. J. Appl. Pharm. 2019; 11: 237–245.
35. Winarti RBS: Antioxidant activity os Sonneratia caseolaris mangrove leaf extract based on leaf maturity level. J. Marine Coastal Sci. 2019; 8(3): 134–136.
36. Morris CJ, Della CD: Using molecular docking and molecular dynamics to investigate protein-ligand interactions. Mod. Phys. Lett. B. 2021; 35: 2130002.
37. Xin X, Tingting Y, Xiang Li, et al.: Recent advances in targeting the “undruggable” proteins: from drug discovery to clinical trials. Sig. Transduct. Target. Ther. 2023;8:335.
38. DeJulius CR, Dollinger BR, Kavanaugh TE, et al.: Optimizing an Antioxidant TEMPO Copolymer for Reactive Oxygen Species Scavenging and Anti-Inflammatory Effects in Vivo. Bioconjug Chem. 2021; 32(5):928-941.
39. Le DQ, Kuriakose AE, Nguyen DX, et al.: Hybrid Nitric oxide donor and its carrier for the treatment of peripheral arterial diseases. Sci. Rep. 2017; 7: 1–11.
40. Hassanpour S, Rezaei H, Razavi SM: Anti-nociceptive and antioxidant activity of betaine on formalin- and writhing tests induced pain in mice. Behav. Brain Res. 2020; 390: 112699.
41. Oh YS, Jun HS: Effects of glucagon-like peptide-1 on oxidative stress and Nrf2 signaling. Int. J. Mol. Sci. 2018; 19(1): 1–16.
42. Hartati K, Fitriana N, Anggraeni IL, et al.: The potential of Sonneratia caseolaris mangrove leaves extract as a bioactive food ingredient using various water extract. [Dataset]. figshare. 2024.

**4. Reviewer's Comment:**

 In the method, the author did not mention the weight of the leaves and the dry leaves and why you chose the leaves from the third to the fifth leaf. 

*Author response:*

 Thanks for your review, we are going to revise it.

**Sample extraction**

 The extraction process utilized various solvent, including distilled water (A), bottled drinking from three different brands (Ax (B), Cx (C), Kx (D), and methanol (Cat. 179337, Sigma-Aldrich ® ) (E) for control purposes. The bottle drinking water

 (B, C, and D) was sourced from the Indonesian market. Mangrove leaf powder was extracted with each solvent at ratio 1:3 (w/v). The extraction was conducted through maceration at room temperature for 24 hours, followed by filtration using

 Whatman ® quantitative filter paper, ashless, Grade 42 (Cat. WHA1442041, Merck). The extraction process was repeated three times for each solvent. The resulting filtrates were subsequently evaporated using a rotary evaporator (DLab rotary

 evaporator RG100-S, 40°C). Every 1000 grams of fresh leaves will become 200 grams of dried leaves. In this study, the third and fifth leaves are selected based on previous research findings. These leaves have shown higher total phenol, total alkaloid, and flavonoid content compared to other parts of the leaf. However, there is no significant difference in pH and viscosity.

**5. Reviewer's Comment:**

 Some sentences will cause misunderstanding, such as whether the author used the dry extract or fractionation to analyze the total phenol and flavonoids. In that section, you used 60 μL and 1 ml samples respectively, but you did not mention the dilution, using what kind of solution you diluted the dry extract.

*Author response:*

 Thanks for your review, we are going to revise it.

**Calculation of extraction yield and pH examination**

 The extraction yield was calculated by the following formula.1 The pH of the extracts was measured with a pH meter

 (Toledo S-220-KIT). The analyzed sample is a working solution derived from the evaporator, consisting of 1 mL of solution mixed with 20 mL of double-distilled water.

**6. Reviewer's Comment:**

 The author did not mention the statistical test they used to get the p-value.

*Author response:*

 The statistical test used was the Duncan Test with SPSS version 21, where the significance level was set at p-value < 0.05. This information will be added as a note in Table 1.

**7. Reviewer's Comment:**

 “Among these, Tempo, Betaine, and Choline were identified as active compounds known for their antioxidant properties” results section, 2nd paragraph line 5. If this statement is from other papers please put the citation. However, it’s better to add biological function analysis of those compounds regarding their antioxidant properties using analysis tools (e.g. PASS online or others) to make this article more enriched.

*Author response:*

 Thanks for your review, we are going to revise it.

 “The content of bioactive compounds in mangrove leaf extract of S. caseolaris was analyzed using LC-HRMS. The analysis results revealed the presence of 16 identical active compounds in all *S. caseolaris* mangrove leaf extracts obtained with different solvents (Table 2). The bioactive compound content in mangrove leaf extract from *S. caseolaris* was assessed through LC-HRMS. The findings from the analysis indicated the presence of 16 identical active compounds in all S. caseolaris mangrove leaf extracts, regardless of the solvents used (refer to Table 2). Among these, Tempo, Betaine, and Choline were identified as active compounds known for their antioxidant properties ^38,40^.”

 We also added some information for biological function of Tempo, Betaine, and Choline, namely:

 “Tempo finds extensive use in cellular and animal model studies due to its favorable characteristics, including low molecular weight, high water solubility, and the capability to permeate cell membranes efficiently ^43^. Betaine, a natural compound, is a glycine derivative with three additional methyl groups, as depicted in Figure 1 in both 2D and 3D forms. It is widely found in plants, animals, and microorganisms, including beets, spinach, wheat bran, wheat germ, and aquatic invertebrates. Betaine can be produced endogenously through choline metabolism or obtained exogenously through dietary intake, exhibiting similar bioavailability whether consumed as a supplement or through food, ultimately metabolizing to dimethylglycine and sarcosine in kidney and liver cells' mitochondria44.

 Choline, a vital nutrient, plays a fundamental role in diverse biological functions such as the synthesis of structural lipoproteins and membrane lipids, neurotransmitters, and the maintenance of brain function. Betaine, derived from choline oxidation, is crucial for the formation of essential amino acids like methionine. Dimethylglycine (DMG), a betaine metabolite, serves as a vital source of glycine and methyl groups for biochemical reactions. Additionally, trimethylamine N-oxide (TMAO) is metabolized in the liver from trimethylamine (TMA), primarily generated from dietary choline and betaine, and emerges as a risk factor in various diseases, including renal disease, cardiovascular disease, colorectal cancer, type II diabetes, and neurological disorders. This highlights the multifaceted role of choline metabolism and its metabolites in health and disease ^45^.

 Reference:

 43. Marcin L, Krzysztof G. 2017. Nitroxides as antioxidants and anticancer drugs. IJMS, 18(11): 2490.

 44. Dobrijević D, Pastor K, Nastić N, et al. J. Betaine as a functional ingredient: metabolism, health-promoting attributes, food sources, applications and analysis methods. Molecules. 2023;28(12):4824.

 45. Yvonne M, Christiane S, Manfred W, Petter-Arnt H. Plasma kinetics of choline and choline metabolitesafter a single dose of superbaboosttm krill oil orcholine bitartrate in healthy volunteers. Nutrients. 2019;11;2548.

**8. Reviewer's Comment:**

 The aim of this study was to evaluate the effectiveness of the bioactive compounds in *S. caseolaris* leaf extract in inhibiting the Keap1 protein. However, I didn’t get the answer about the effectiveness of those compounds compared to the inhibitor. 

*Author response:*

 Thanks for your review, we are going to revise it.

 “We aim to enhance our analysis to predict how potential compounds in *Sonneratia caseolaris* leaves water extract interact with the target protein Keap1. *S. caseolaris*, has potential compounds interact with a target protein like Keap1, is crucial for understanding their potential therapeutic potential.”

 -------------------------------------

 We believe that implementing these changes has strengthened the manuscript and enhanced its contribution to the field. We are confident that the revised version now meets the standards of F1000 and will be of interest to its readership.

 Once again, we appreciate your time and effort in reviewing our manuscript. Your expertise and constructive criticism have been invaluable in improving our work. Please do not hesitate to contact us if you have any further questions or require additional clarification.

 Thank you for your consideration.

 Sincerely,

 Hartati Kartikaningsih

 Universitas Brawijaya
